# Outcomes of Pediatric Patients With Sepsis Related to *Staphylococcus aureus* and Methicillin-Resistant *Staphylococcus aureus* Infections Requiring Extracorporeal Life Support: An ELSO Database Study

**DOI:** 10.3389/fped.2021.706638

**Published:** 2021-10-08

**Authors:** Cortney Foster, Dayanand Bagdure, Jason Custer, Adrian Holloway, Peter Rycus, Jenni Day, Adnan Bhutta

**Affiliations:** ^1^Division of Pediatric Critical Care, Department of Pediatrics, University of Maryland, Baltimore, MD, United States; ^2^ELSO, Ann Arbor, MI, United States; ^3^Department of Nursing and Patient Care Services, University of Maryland Medical Center, Baltimore, MD, United States

**Keywords:** ECMO, MSSA, MRSA, outcomes, sepsis, pediatric

## Abstract

**Background:** Extracorporeal membrane oxygenation (ECMO) is increasingly utilized for pediatric sepsis unresponsive to steroids and inotropic support. Outcomes of children with sepsis are influenced by the type of pathogen causing their illness.

**Objective:** To determine if the outcomes of children with *Staphylococcus aureus* sepsis receiving ECMO differed according to microbial sensitivity (Methicillin-resistant *Staphylococcus aureus* [MRSA] vs. Methicillin-sensitive *Staphylococcus aureus* [MSSA]).

**Methods:** Retrospective case-matched cohort study of children (0–<18 years) with *Staphylococcus aureus* sepsis reported to the ELSO registry from more than 995 centers. Inclusion criteria were age 0–18 years, laboratory diagnosis of Staphylococcal infection, clinical diagnosis of sepsis, and ECMO deployment. Exclusion criteria were no laboratory diagnosis of Staphylococcal infection. We compared patient demographics, pre-ECMO management and outcomes of those with MRSA vs. MSSA using Chi-Square test, with independent samples *t*-test used to test to compare continuous variables.

**Results:** In our study cohort of 308 patients, 160 (52%) had MSSA and 148 (48%) MRSA with an overall survival rate of 41.5%. There were no differences in the age group (*p* = 0.76), gender distribution (*p* = 0.1) or racial distribution (*p* = 0.58) between the two groups. *P* value for racial distribution should be 0.058. There were 91 (56.8%) deaths in the MSSA group and 89 (60.1%) deaths (*p* = 0.56) in the MRSA group. Duration on ECMO (*p* = 0.085) and the time from intubation to ECMO (*p* = 0.37) were also similar in the two groups. Survival with MSSA sepsis and MRSA sepsis did not improve significantly over the 20 years evaluated despite an increase in ECMO utilization.

**Conclusion:** In this multi-center retrospective study, there were no differences in outcomes for children receiving ECMO support with *Staphylococcus aureus* sepsis according to microbial methicillin sensitivity. There was no significant increase in survival among patients with MRSA and MSSA infections receiving ECMO in the last 20 years.

## Introduction

Severe sepsis and septic shock remain leading causes of pediatric mortality globally (1). Sepsis in children accounts for >75,000 annual admissions in the United States and has an estimated mortality rate of 5–20% (2). Age, cardiovascular comorbidity, and organ dysfunction are associated with increased mortality risk (3). The majority of children who die of sepsis experience multi-organ dysfunction and refractory shock and often do so within the first 72 h of their hospital admission (4).

*Staphylococcus aureus* is a common cause of sepsis and septic shock in the pediatric population. Methicillin-resistant *Staphylococcus aureus* (MRSA) is a virulent pathogen with high mortality (5). The virulence patterns of MRSA can affect outcomes of patients infected with this organism. A meta-analysis of the differences in mortality between pediatric patients with MRSA and methicillin sensitive *Staphylococcus aureus (*MSSA) bacteremia found a significantly higher mortality risk (OR = 2.33) in patients with MRSA bacteremia than in those with MSSA bacteremia. Specifically, there was a mean mortality rate of 9% in the MSSA group compared to 20.9% in the MRSA group (6).

Many strides have been made in the management of pediatric severe sepsis in the last 10 years, including the adjuvant use of extracorporeal therapies (7). A 2011 review of Extracorporeal Life Support Organization (ELSO) database found an overall survival rate of 68% in pediatric patients with severe sepsis requiring ECMO (8), compared to previous survival data of 38.6% (9). The pediatric Surviving Sepsis Campaign 2020 guidelines include the consideration of veno-arterial ECMO as a rescue therapy in children with refractory septic shock (4).

There are limited data on the outcomes of pediatric patients with severe sepsis from MSSA and MRSA who require management with ECMO. We hypothesized that patients with MRSA sepsis and septic shock receiving ECMO would have worse outcomes than patients with MSSA sepsis and septic shock receiving ECMO. We also compared survivors and non-survivors within each group, with a hypothesis that survivors would have better respiratory and physiologic parameters than non-survivors.

## Materials and Methods

This retrospective case cohort analysis was approved by the ELSO Registry Scientific Oversight Committee and exempted from our local IRB. The ELSO registry includes descriptive clinical data from patients who receive ECMO from 995 international centers. Study population was identified by age (0–<18 years) between years 1996 and 2015 and diagnostic ICD-9 codes associated with sepsis and septic shock (785.52, 995.92, 995.91, 995.9, 040.82, 785.5, 038.1). We matched patients to those who had a culture-isolated *Staphylococcus aureus* (MRSA or MSSA) either pre-ECMO or during ECMO. Patients with positive cultures only after ECMO were not included in the analysis. The analysis included only first ECMO run for each patient.

Data were analyzed using SPSS-25. Descriptive statistics were calculated to describe the sample. Data were evaluated for completeness and no participants were missing >50% of data. No data imputation method was used. A Chi-Square test was used to test for differences between bacterial infection type (MRSA and MSSA) and other categorical variables (survival outcome, gender, race, mode, support type). Continuous variables were tested to assure they met the assumptions for parametric statistics and we used an independent samples *t*-test to test for differences in continuous variables (age, ECMO time, intubation time to ECMO, ventilator settings, blood gas values, blood pressure) and bacterial infection type (MRSA and MSSA). Multiple logistic regression models were used to evaluate the impact of different factors on survival. Factors were separated into physiologic and respiratory-support related factors. A multiple linear regression was calculated to see if bacterial infection type (MRSA and MSSA) and epochs (5 year ranges 1996–2015) predicted survival rate. Logistic regression was performed across groups to assess the impact of infection type on the odds that pediatric patients would survive following ECMO for severe sepsis. The model controlled for time, age (days), intubation to ECMO time (hours), and support type (pulmonary, cardiac, ECPR) and contained infection type as the independent variable.

Separate analyses were conducted for both MRSA and MSSA, and factors were separated into physiologic and respiratory-support related factors. Logistic regression analyses were performed to assess the impact of a set of physiologic predictors on the odds that pediatric patients would survive following ECMO for severe sepsis. The model controlled for time, age (days), intubation to ECMO time (hours), and support type (pulmonary, cardiac, ECPR) and contained pH, pCO_2_, pO_2_, HCO_3_, SaO_2_, SBP, DBP as the independent variables. Logistic regression was also performed to assess the impact of a set of respiratory support-related predictors on the odds that pediatric patients would survive following ECMO for severe sepsis. The model controlled for time, age (days), intubation to ECMO time (hours), and support type (pulmonary, cardiac, ECPR) and contained ECMO time (in hours), VV/VA mode, initial settings (FiO2, PIP, PEEP, MAP) and settings at 24 h (FiO_2_, PIP, PEEP, MAP) as the independent variables. The *p*-value was set at 0.05 for statistical significance.

## Results

A total of 308 patients met study inclusion criteria. Of these, 160 (51.9%) had MSSA-related sepsis and 148 (48%) had MRSA sepsis. Demographic data are included in Table 1. There were no significant differences in demographics between those with MSSA vs. those with MRSA sepsis. A total of 229 patients were managed on VA ECMO and 71 patients were managed on VV ECMO. There was no difference in type of ECMO delivery between the groups (*X*^2^ = 1.002, *p* = 0.317).

**Table 1:** Characteristics of children receiving ECMO due to MSSA or MRSA sepsis.

**Variable**	**MSSA**	**MRSA**	**Statistical test**		
	**N 160**		**N 148**		
	***M* (SD)**	***N* (%)**	***M* (SD)**	***N* (%)**	
Age (years)	8.02 (6.11)		8.23 (6.34)		*t* = −0.294, df = 306, *p* = 0.769
**Gender**					
Male		82 (51)		89 (60)	*X*^2^ (1, *N* = 307) = 2.682, *p* =0.101
Female		78 (49)		58 (39)	
**Race**					
White		94 (59)		87 (59)	*X*^2^ (4, *N* = 305) = 9.125, *p* = 0.058
Black		32 (20)		31 (21)	
Hispanic		14 (9)		21 (14)	
Asian		9 (5)		4 (3)	
Other		11 (7)		2 (1)	
Duration on ECMO (days)	10.8 (10.7)		13.4 (14.4)		*t* = 1.730, df = 301, *p* = 0.085
Intubation to ECMO (days)	4.7 (11.3)		3.7 (7.5)		*t* = −0.882, df = 294, *p* = 0.379
**Outcome**					
Survived		69 (43)		59 (40)	*X*^2^ (1, *N* = 308) = 0.336, *p* = 0.562
Died		91 (57)		89 (60)	
MAP		24.4 (8.3)		27.8 (9.8)	0.005
MAP at 24 h		14.7 (5.3)		16.7 (5.9)	0.008
PEEP at 24 h		9.7 (2.7)		11.1 (4.3)	0.005
**Support mode**					
VA		122 (76)		107 (72)	*X*^2^ (1, *N* = 300) = 1.002, *p* = 0.317
VV		33 (21)		38 (26)	

Overall survival of all patients was 41.5% (MSSA 43.1% and MRSA 39.8%, *X*^2^ = 0.336, *p* = 0.56). There was no difference in patient outcome based on support type (*X*^2^ = 3.293, *p* = 0.193); 217 patients were supported for pulmonary failure, 52 patients were supported due to cardiac failure and 39 patients were extracorporeal cardiopulmonary resuscitation cases. Duration on ECMO and the time from intubation to ECMO were similar in the MSSA and MRSA groups. Patients with MRSA had significantly higher pre-ECMO mean airway pressures than those with MSSA sepsis (27.8 vs. 24.4, *p* = 0.005). In addition, patients with MRSA continued with higher mean airway pressure and positive end-expiratory values at 24 h post ECMO initiation (16.7 vs. 14.7, *p* = 0.008 and 11.1 vs. 9.7, *p* = 0.005) (see Table 1). Logistic regression to assess the impact of a set of physiologic predictors on the odds that pediatric patients would survive following ECMO for severe sepsis was not statistically significant *X*^2^ (8, *N* = 183) = 11.586, *p* =0.171.

Logistic regression was performed across groups to assess the impact of a set of respiratory support-related predictors on the odds that pediatric patients would survive following ECMO for severe sepsis. The model controlled for time, age (days), intubation to ECMO time (hours), and support type (pulmonary, cardiac, ECPR) and contained infection type, ECMO time (in hours), VV/VA mode, initial settings (FiO_2_, PIP, PEEP) and settings at 24 h (FiO2, PIP, PEEP) as the independent variables. The full model was not statistically significant *X*^2^ (8, *N* = 108) = 8.361, *p* = 0.399.

For pediatric patients with sepsis and a positive MSSA culture, the full logistic regression model to assess the impact of physiologic predictors on the odds that pediatric patients would survive following ECMO for severe sepsis was not statistically significant *X*^2^ (15, *N* = 122) = 17.908, *p* = 0.268. Also for the MSSA group, the full logistic regression model to assess the impact of a set of respiratory support-related predictors on the odds that pediatric patients would survive following ECMO for severe sepsis was statistically significant *X*^2^ (18, *N* = 45) = 31.821, *p* = 0.023. The model explained 68% (Nagelkerke *R*^2^) of the variance in survival and the model as a whole correctly classified 86.7% of cases. The only two variables that made a unique statistically significant contribution to the model were age in days (OR 1.001) and use of VV mode.

Survivors of ECMO with MRSA sepsis had lower positive end-expiratory pressure levels and lower mean airway pressure at 24 h post ECMO initiation compared to those who died of MRSA sepsis (9.9 vs. 12, *p* = 0.006, and 15.4 vs. 17.8, *p* = 0.036). No significant differences were found in fraction of inspired oxygen or peak-inspiratory pressure at 24 h between patients who survived or died with MRSA sepsis. Patients with MRSA sepsis who died were found to have significantly lower pH than those who survived with MRSA sepsis (7.13 vs. 7.19, *p* = 0.017). For pediatric patients with sepsis and a positive MRSA culture, the logistic regression model to assess the impact of physiologic predictors on the odds that pediatric patients would survive following ECMO for severe sepsis was not statistically significant *X*^2^ (14, *N* = 109) = 23.353, *p* = 0.055. When examining the impact of respiratory support-related predictors for the MRSA group on the odds that pediatric patients would survive following ECMO for severe sepsis, the full logistic regression model was not statistically significant *X*^2^ (16, *N* = 34) = 24.044, *p* = 0.089. Respiratory parameter data are included in Table 2.

**Table 2:** Differences in the MSSA and MRSA sepsis cohort on ECMO according to the survival status.

**Variable**	**MSSA (N 160)**		***t***	***p***	**MRSA (N 148)**		***t***	***p***
**Survival status**	**Survived (N 69)**	**Died (N 91)**			**Survived (N 59)**	**Died (N 89)**		
Age (years)	7.8 (6.1)	8.5 (6.5)	−0.799	0.425	6.5 (5.7)	9 (6.2)	–**2.475**	**0.014**
Duration on ECMO (days)	11 (9.1)	10.7 (11.8)	0.194	0.846	14.3 (9.7)	12.7 (16.9)	0.616	0.539
Intubation to ECMO (days)	2.5 (2.98)	6.5 (14.5)	–**2.536**	**0.013**	4.2 (8)	3.5 (7.4)	0.537	0.592
FiO_2_	91.1 (17.2)	94.2 (14.3)	−1.176	0.242	93 (14.1)	95.9 (12.1)	−1.235	0.219
PiP	46.9 (23.1)	43.6 (17.7)	0.904	0.368	48.8 (22.7)	48.9 (20.5)	−0.017	0.987
PEEP	10.9 (5.8)	11.1 (5.1)	−0.208	0.835	12 (5.7)	12.1 (7.4)	−0.075	0.940
MAP	24.4 (8.4)	24.5 (8.4)	−0.062	0.951	26.8 (7.3)	28.6 (11.2)	−0.973	0.333
FiO_2_ at 24 h	42.7 (19.9)	46.3 (18.9)	−1.108	0.270	42.9 (18.7)	49 (20.8)	−1.778	0.078
PiP at 24 h	29.1 (15.1)	28.9 (14.2)	0.039	0.969	28.1 (13.9)	31.4 (13.6)	−1.368	0.174
PEEP at 24 h	9.9 (2.98)	9.6 (2.5)	0.765	0.454	9.9 (3.3)	12 (4.7)	–**2.826**	**0.006**
MAP at 24 h	14.4 (5.5)	15 (76.3)	−0.567	0.572	15.4 (6.4)	17.8 (5.4)	–**2.120**	**0.036**
pH	7.17 (0.16)	7.13 (0.18)	1.168	0.245	7.19 (0.15)	7.13 (0.17)	**2.409**	**0.017**
pCO_2_	60.3 (23.4)	65.8 (27.8)	−1.301	0.195	65.3 (27.7)	71.9 (29.7)	−1.335	0.184
pO_2_	79.1 (59.8)	63.8 (36.9)	1.818	0.072	69.1 (36.8)	82.4 (83.7)	−1.285	0.201
Sats	81.7 (15.5)	76.3 (21.9)	1.752	0.082	81.1 (17.7)	74.2 (23.7)	1.915	0.058
SBP	79.1 (26)	75.5 (25.7)	0.818	0.414	81.4 (26.8)	76.5 (26)	1.052	0.295
DBP	43.6 (13.1)	40.8 (13.1)	1.280	0.203	45.7 (17.6)	42.1 (14.6)	1.262	0.209

*The bolded are the only statistically significant values*.

**Figure 1:**
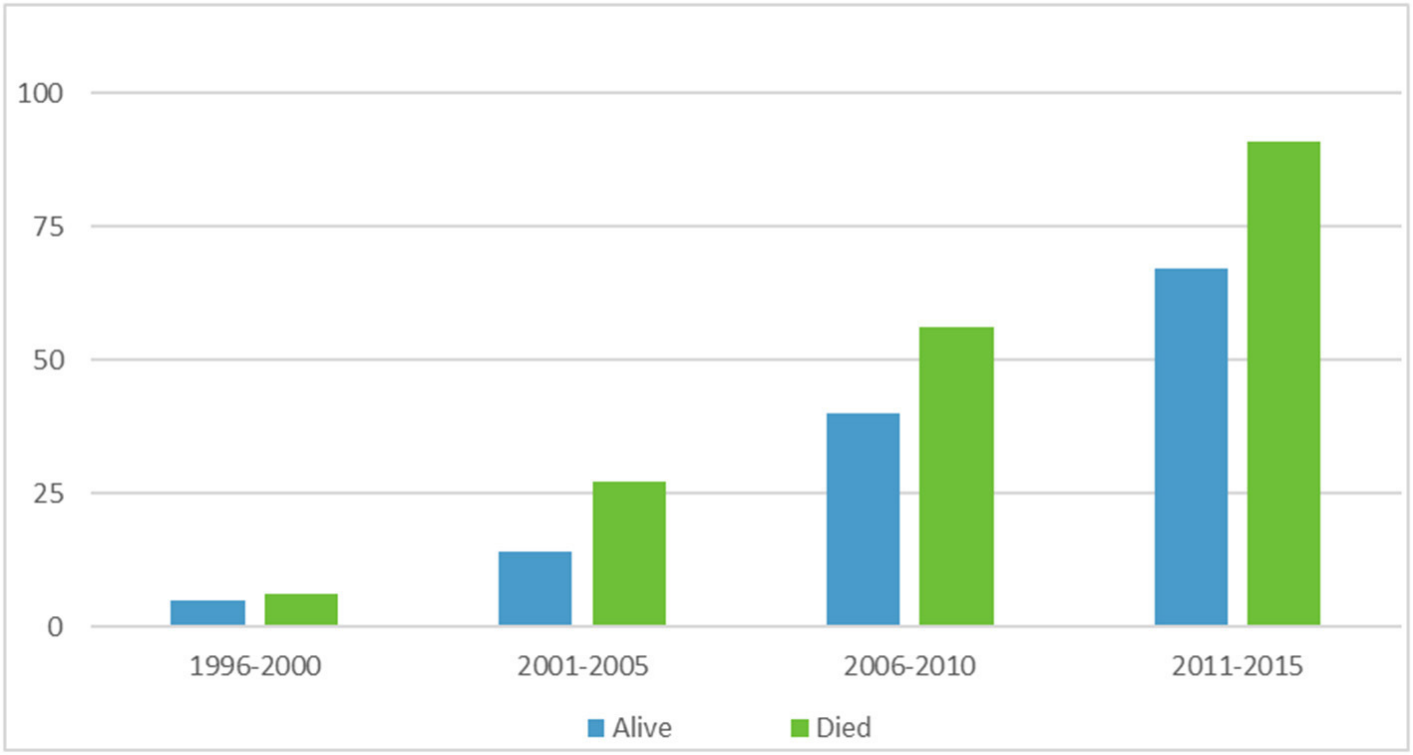
ECMO utilization and survival in 5 year increments.

**Figure 2:**
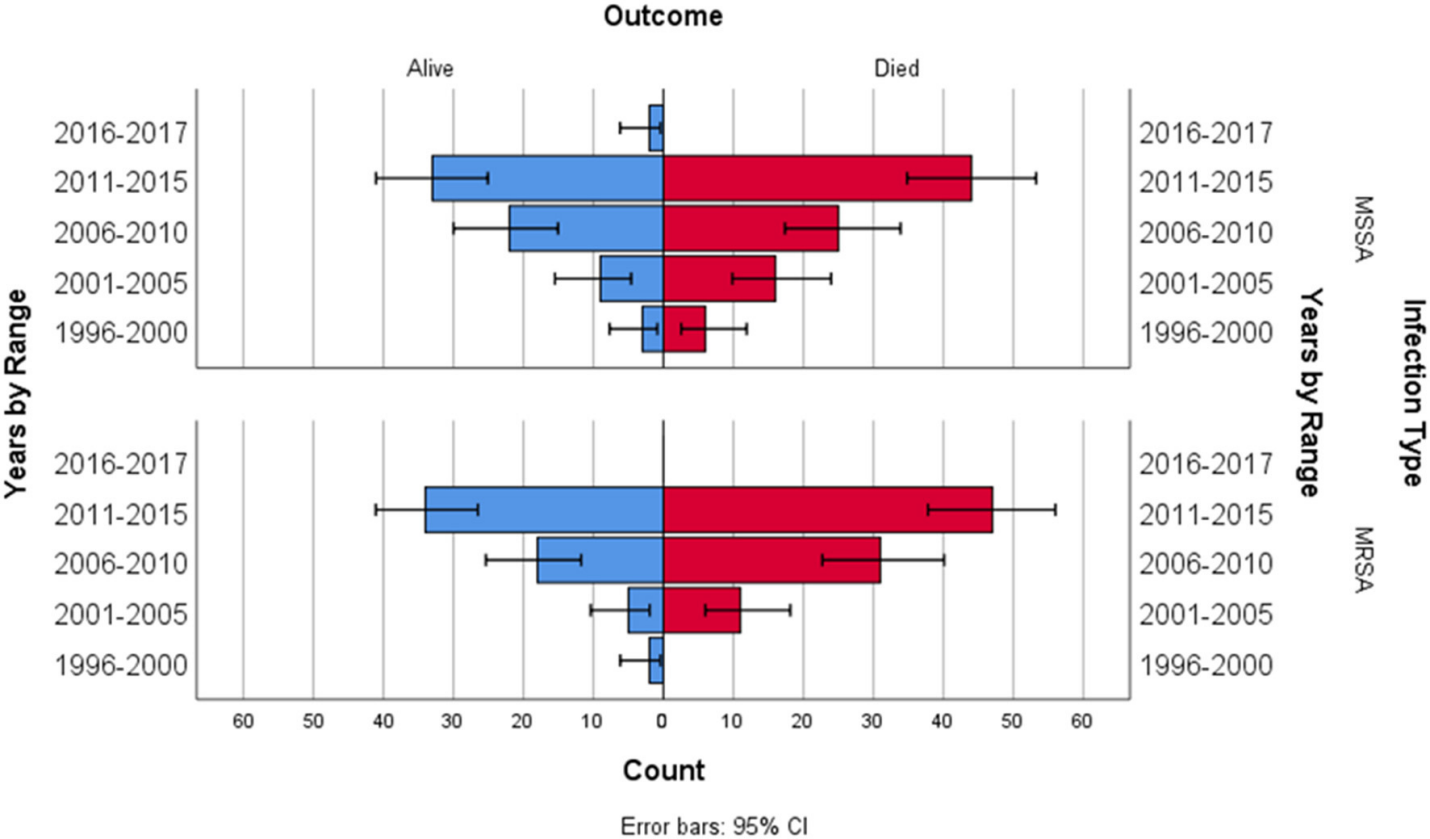
Outcome by year and infection type.

A multiple linear regression was calculated to see if bacterial infection type (MRSA and MSSA) and epochs (5 year ranges 1996–2015) predicted survival rate. In the multiple linear regression model, a non-significant regression equation was found [*F*_(3,4)_ = 1.431, *p* = 0.358], with an *R*^2^ = 0.518. Figure 1 depicts ECMO utilization in 5 year increments, Figure 2 depicts outcome by year and infection type.

## Discussion

Despite a difference in pathogenicity, we did not see a significant difference in outcomes for children with MSSA or MRSA sepsis receiving ECMO. The overall survival rate of patients with MSSA and MRSA sepsis receiving ECMO therapy in this study was 41.5%, which is similar to the 39.4% survival of all septic pediatric patients on ECMO from 1996 to 2015. A review of pediatric ECMO from 2009 to 2015 found an overall survival rate of 61% (10). A review of the ELSO registry from 1990 to 2008 evaluated pediatric non-cardiac septic patients receiving ECMO and found an overall survival of 68% (8).

Survival of MRSA and MSSA patients did not significantly improve over the years evaluated in our study. The duration on ECMO and the time from intubation to ECMO were similar in the two groups. This lack of a difference in mortality and days on ECMO could be due to the presence of circulatory support, aggressive use of antibiotics and adherence to sepsis management guidelines.

Patients with MRSA had significantly higher mean airway pressures than those with MSSA sepsis as well as higher positive end-expiratory values and mean airway pressure levels at 24 h post ECMO initiation compared to MSSA patients. This difference in airway pressure could be related to a worsened severity of illness and lung disease in the MRSA group compared to the MSSA group. Despite these differences in positive end-expiratory and mean airway pressure at 24 h, there were no significant differences in survival in these groups. An additional finding in our study was that patients who survived with MRSA sepsis had lower positive end-expiratory levels at 24 h post ECMO initiation compared to those who died of MRSA sepsis. In addition, patients who survived with MRSA sepsis had lower mean airway pressure at 24 h post ECMO initiation compared to those who died of MRSA sepsis. These findings could be related to severity of lung disease in the patients who died with MRSA sepsis. It could also be related to improved lung protective mechanisms in the survival group post initiation of ECMO. Overall, the patients in our study had very high peak inflating pressures pre ECMO and they maintained high peak inflating airway pressures at 24 h post ECMO initiation, as well as high driving pressures (plateau pressure minus positive end-expiratory pressure). It is unknown if patients were maintained in a pressure mode or volume mode. A recent retrospective cohort study found that a driving pressure <15 cm H_2_O was associated with significantly decreased morbidity in children with acute hypoxemic respiratory failure (11).

We did find that patients who survived with MSSA sepsis tended to receive ECMO more quickly after intubation compared to those who died with MSSA sepsis. Earlier ECMO initiation may help to mitigate or more quickly reverse organ dysfunction. Although there are no pediatric data to support this hypothesis, adult studies suggest improved survival with earlier initiation of support. A study by Cheng on adult ECMO for sepsis found that initiation of ECMO within 96 h from time of admission was associated with improved survival when compared to later support (12). Center size and volume may also play a role in survival. It is possible that larger centers are able to initiate ECMO more quickly and also have more expertise in ECMO management, thus contributing to survival. An evaluation of the Pediatric Health Information System database from 2004 to 2011 found increased mortality in centers with low extracorporeal membrane oxygenation average annual case volume (13). A single center study by MacLaren et al. employed central ECMO for refractory septic shock and had a 78% survival off of ECMO and 74% survival to hospital discharge (14). Survival may also be impacted by support type. Even though there was no difference in survival across groups depending on support type, we did find that within the MSSA group, patients who were managed on VV ECMO were more likely to survive than those managed by VA ECMO. This finding is supported by the review of the ELSO registry from 1990 to 2008 which found improved survival in VV ECMO for non-cardiac sepsis compared to VA ECMO (8).

This study has some important limitations. Given the retrospective nature of the study it is possible that confounding variables were missed in this analysis. In addition, the ELSO registry does not include data from all centers in the world, only centers that are participating. This may limit the generalizability of results. Given the use of the database for data extraction, we were unable to access variables that may have been significant clinically. We were limited in data that evaluated multi-organ dysfunction, as well as other markers of severity of illness of patients. It is possible that there were differences in outcome measures including organ failure, ventilator-free days, need for dialysis, and degree of lung injury, that we did not investigate in this study. In addition, the use of the ICD-9 codes may have limited our access to data as they are subject to coding error and misclassification and may lead to under diagnosis and missed cases. Also, differences in ventilator settings at 24 h may be related to local institutional ventilator weaning practices. It is difficult to interpret these 24 h post-ECMO ventilator data without knowing individual institutional practices which are not available in database studies. An additional limitation of our study is regarding the retrospective database nature of the study. Patients were included when they had a diagnostic code for septic shock or sepsis before ECMO deployment or during their ECMO course, and also had a positive blood culture with *Staphylococcal* species during that time. It is possible that another organism that was not detected could have contributed to their sepsis.

In conclusion, we found no significant differences in outcomes of children with MSSA and MRSA sepsis requiring ECMO. We did find hemodynamic and ventilator settings that may have an impact on the outcomes of children requiring ECMO, warranting further studies to evaluate other outcomes measures. We did not find an increase in survival in patients over the years evaluated, despite an increase in ECMO utilization.

## Data Availability Statement

The data analyzed in this study is subject to the following licenses/restrictions: You must be a member of ELSO registry. Requests to access these datasets should be directed to prycus@elso.org.

## Author Contributions

All authors listed have made a substantial, direct and intellectual contribution to the work, and approved it for publication.

## Conflict of Interest

The authors declare that the research was conducted in the absence of any commercial or financial relationships that could be construed as a potential conflict of interest.

## Publisher's Note

All claims expressed in this article are solely those of the authors and do not necessarily represent those of their affiliated organizations, or those of the publisher, the editors and the reviewers. Any product that may be evaluated in this article, or claim that may be made by its manufacturer, is not guaranteed or endorsed by the publisher.
